# Stratified obstetric management for heterogeneous rare diseases: a precision medicine framework based on four genetic archetypes

**DOI:** 10.3389/fmed.2026.1820341

**Published:** 2026-05-08

**Authors:** Shangling Lv, Haipeng Yang, Yuying Cui, Haiyan Yang

**Affiliations:** 1 Department of Obstetrics, Yantai Yantaishan Hospital, Yantai, Shandong, China; 2 Yantai Qishan Hospital, Yantai, Shandong, China; 3 Aier Eye Hospital, Yantai, Shandong, China

**Keywords:** expanded carrier screening, maternal-fetal medicine, noninvasive prenatal testing (NIPT), precision medicine, preimplantation genetic testing, prenatal diagnosis, rare diseases

## Abstract

Rapid advances in genomic sequencing increasingly expose obstetricians to complex and heterogeneous genetic information in routine clinical practice. Traditional disease-specific screening models are no longer sufficient to guide decision-making across the broad spectrum of rare disorders encountered in reproductive medicine. Frontline clinicians require a universal yet clinically intuitive framework that links the biological logic of disease to the most appropriate obstetric intervention. In this review, we selected seven representative rare diseases as clinical models and reorganized them into four genetic archetypes according to inheritance pattern, age of onset, testability, and maternal-fetal risk. These archetypes illustrate distinct management pathways: targeted mid-trimester interception of *de novo* dominant mutations, preconception prevention of inherited late-onset tumor syndromes, early defense against highly heterogeneous recessive disorders, and recognition of current predictive blind spots in complex polygenic disease. We further emphasize that some genetic conditions pose direct and potentially life-threatening risks to the pregnant patient and therefore require a maternal protection strategy in parallel with fetal evaluation. This archetype-based framework is intended to facilitate communication between obstetricians and geneticists, improve the clinical interpretation of genomic findings, and provide a practical blueprint for precision obstetric management in the genomic era.

## 1 Introduction

Clinical obstetrics is undergoing a profound genomic transition. With the increasing integration of high-throughput sequencing into reproductive medicine, obstetricians are now confronted with a growing spectrum of rare and ultra-rare disorders during preconception counseling, prenatal screening, and maternal-fetal risk assessment (1, 2). Although each individual rare disease is uncommon, their collective burden in reproductive care is substantial, and the resulting genomic information is often difficult to translate into clear obstetric decisions.

Existing recommendations from professional societies, including the American College of Medical Genetics and Genomics and the American College of Obstetricians and Gynecologists, provide valuable guidance for selected carrier screening scenarios, prenatal diagnosis, and variant interpretation (3, 4). However, these recommendations are largely condition-specific or test-specific. In routine clinical practice, frontline obstetricians frequently face highly heterogeneous findings, including *de novo* pathogenic variants, late-onset hereditary cancer syndromes, genetically heterogeneous recessive disorders, and complex polygenic conditions that remain beyond current prenatal predictive capacity. What remains lacking is a clinically intuitive, cross-disease framework that links underlying genetic architecture to the most appropriate obstetric intervention.

This need is further underscored by emerging population studies showing marked geographic heterogeneity in recessive disease burden and carrier distribution. Recent data from Western Romania demonstrated a high burden of autosomal recessive carrier status in a reproductive-age population (5), whereas an expanded carrier screening study from southern and southwestern China similarly identified a substantial frequency of pathogenic or likely pathogenic variants in apparently healthy individuals (6). These observations suggest that reproductive genetic risk cannot be adequately addressed through static or regionally narrow disease lists alone, but instead requires a framework that accommodates both allelic diversity and differences in disease architecture across populations.

In this review, we do not attempt to compile an exhaustive catalog of rare diseases relevant to obstetric practice. Instead, we select seven representative disorders as clinical models and reorganize them into four genetic archetypes according to inheritance mechanism, age of onset, testability, and maternal-fetal risk. On this basis, we propose a stratified precision-medicine framework intended to help obstetricians and medical geneticists align specific genomic tools—including targeted noninvasive prenatal testing for single-gene disorders, preimplantation genetic testing, expanded carrier screening, invasive prenatal diagnosis, and exclusionary sequencing strategies—with the biological logic of the disease under consideration.

## 2 Conceptual framework for stratified obstetric management

**Figure 1:**
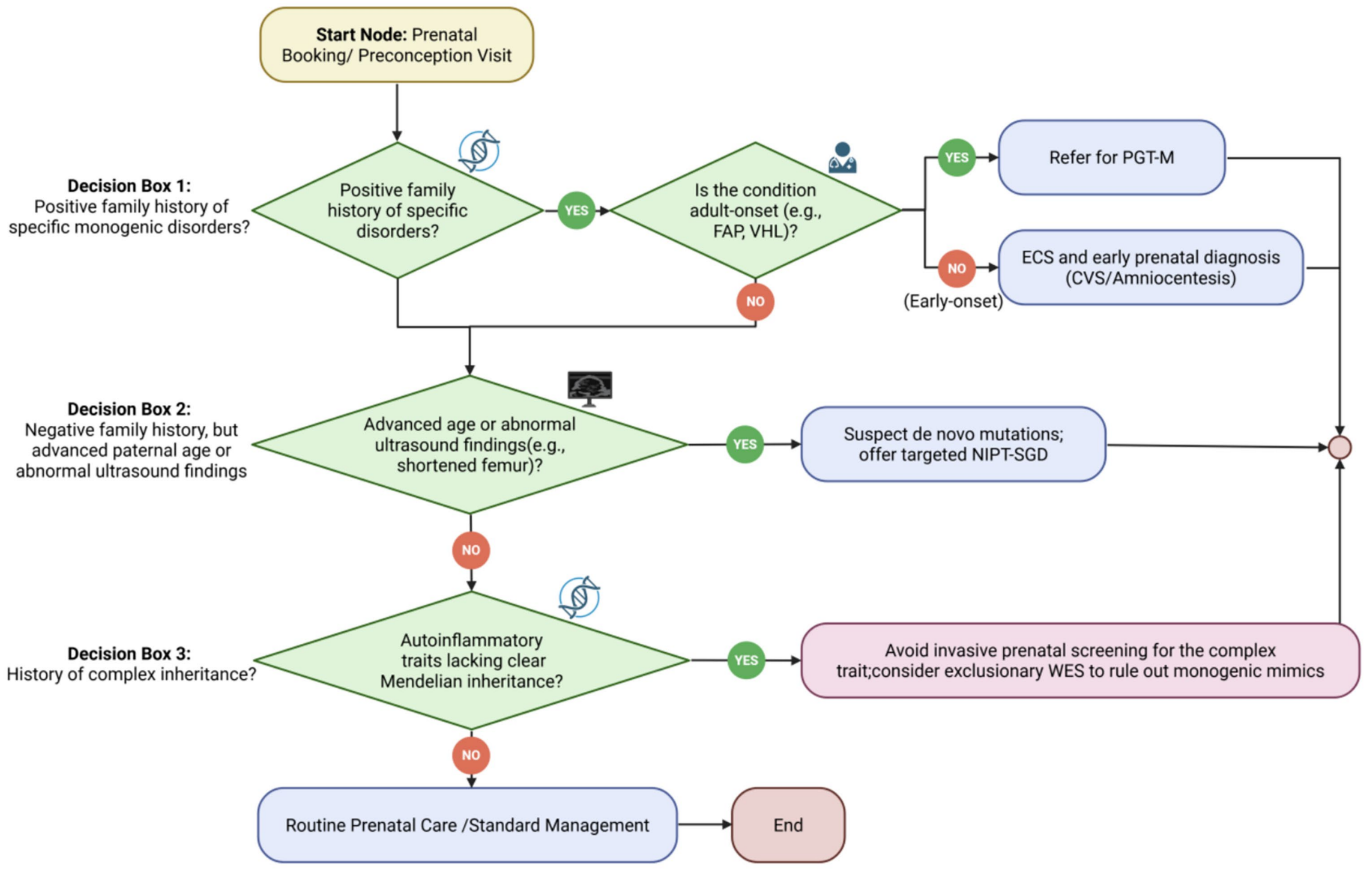
Algorithm for prenatal genomic screening and intervention. A step-by-step clinical triage algorithm designed for frontline obstetricians. The flowchart navigates through routine clinical presentations, such as family history, advanced paternal age, and abnormal ultrasound findings, to appropriately direct patients toward preimplantation genetic testing (PGT-M), targeted noninvasive prenatal testing (NIPT-SGD), or expanded carrier screening (ECS), while explicitly defining the boundaries where prenatal diagnostic testing is contraindicated (e.g., complex polygenic traits).

Precision obstetric management should not be structured solely around individual disease entities, but rather around recurring genetic architectures and the clinical decision patterns they generate. Across the spectrum of rare diseases encountered in reproductive medicine, the most consequential determinants of management are not the labels of specific disorders per se, but whether the condition arises *de novo* or is inherited, whether its phenotypic consequences emerge during fetal life or only later in life, whether it is amenable to targeted molecular interrogation, and whether pregnancy itself imposes direct risk on the mother. This conceptual shift from disease enumeration to mechanism-based stratification forms the foundation of the proposed framework (Figure 1).

**Figure 2:**
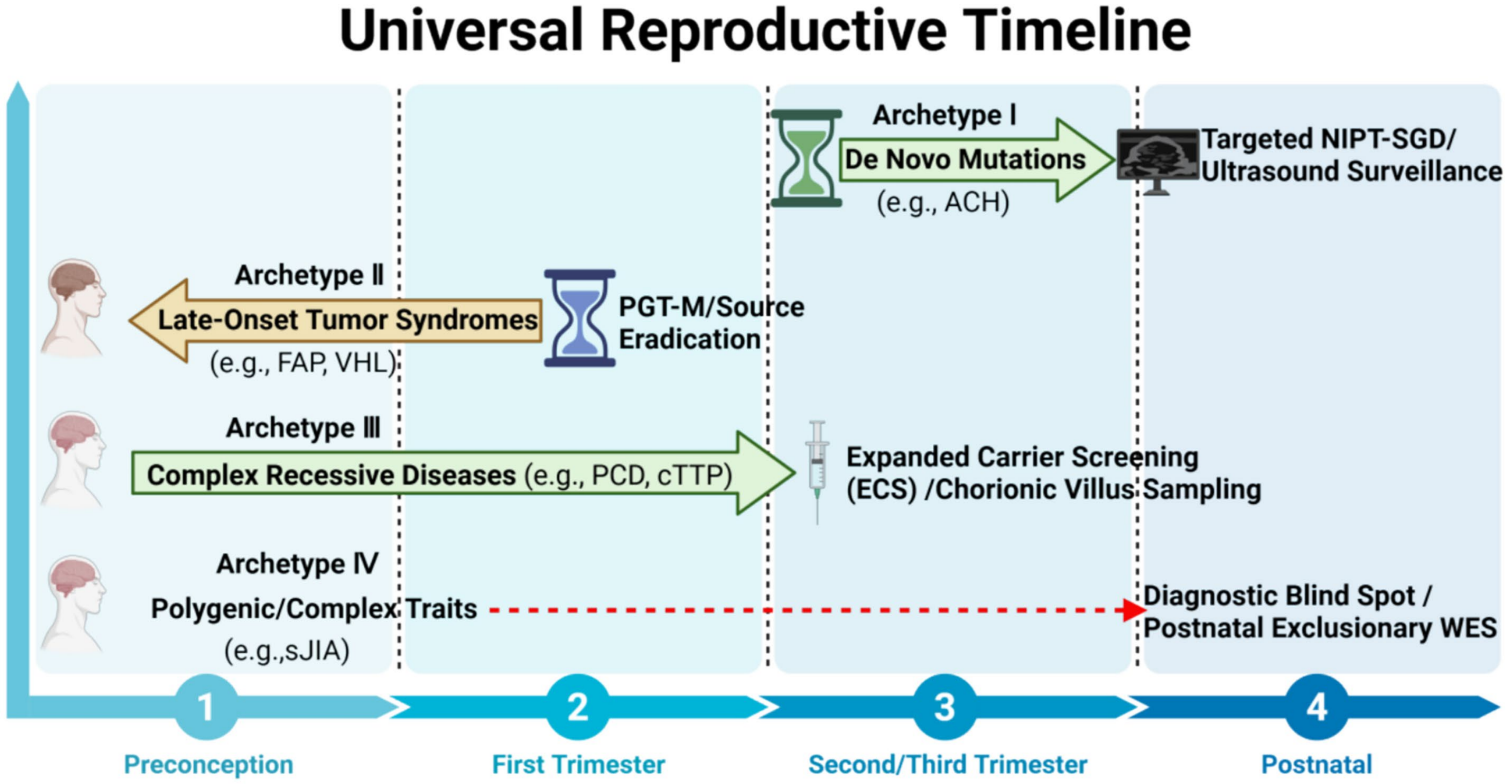
The four genetic archetypes in obstetric precision management. This central framework maps the four primary genetic archetypes to their optimal windows for clinical intervention across the reproductive timeline. Unlike traditional single-disease models, this archetype-based approach highlights that age of onset and underlying genetic architecture (ranging from *de novo* single-point mutations to complex polygenic traits) are the fundamental determinants for selecting the appropriate genomic tool (NIPT, PGT-M, ECS, or exclusionary WES).

On this basis, representative conditions can be organized into four genetic archetypes that capture the major obstetric scenarios created by contemporary genomic medicine (Figure 2; Table 1). Archetype I comprises disorders typically driven by *de novo* dominant variants that are largely invisible to routine parental carrier screening but may be suspected through prenatal imaging and clarified by targeted single-gene analysis. Archetype II includes inherited late-onset disorders, particularly tumor-predisposition syndromes, for which the most ethically and clinically appropriate intervention often lies in preconception embryo selection rather than mid-gestation diagnosis. Archetype III encompasses highly heterogeneous recessive disorders, in which expanded carrier screening followed by confirmatory invasive prenatal diagnosis is generally more informative than narrowly targeted testing. Archetype IV represents polygenic or otherwise currently non-predictable conditions, for which prenatal genomic testing should not be overstated and exclusionary diagnostic reasoning remains the more appropriate clinical approach.

**Table 1:** Characteristics and obstetric management strategies of the seven representative rare diseases categorized by genetic archetype.

Clinical archetype	Disease model	Inheritance pattern	Key genes/Loci	Primary obstetric intervention	Specific maternal risk during pregnancy	Limitations of current prenatal testing
I: *de Novo*	ACH (8, 9)	AD (>80% *de novo*)	*FGFR3*	Mid-trimester US + targeted NIPT-SGD	Minimal maternal risk.	Timing dependent on mid-trimester US findings; NIPT-SGD not routine globally.
I: *de Novo*	OI/NF1 (11, 12)	AD (high de novo rate)	*COL1A1/2, NF1*	Targeted NIPT-SGD/US surveillance	Variable (e.g., NF1 tumors may grow during pregnancy).	Phenotypic variability is high; NIPT-SGD panel coverage varies significantly.
II: Late-Onset	FAP (16)	AD (inherited)	*APC*	Preconception PGT-M	Minimal maternal risk.	Structurally normal fetus; agonizing ethical termination decisions in T2.
II: late-onset	VHL (15, 19)	AD (inherited)	*VHL*	Preconception PGT-M + maternal MDT monitoring	Fatal hypertensive Crisis (triggered by silent pheochromocytoma/labor pain).	Same ethical dilemma as FAP; high risk of maternal mortality if VHL unknown.
III: complex recessive	PCD (20, 21)	AR (high heterogeneity)	Multiple (>50)	ECS + confirmatory invasive prenatal diagnosis (CVS or amniocentesis)	Minimal maternal risk.	Limited by locus heterogeneity; CVS findings may require clarification by amniocentesis if placental mosaicism is suspected.
III: complex recessive	NCL (24)	AR (high heterogeneity)	Multiple (*CLN* genes)	ECS + confirmatory invasive prenatal diagnosis (CVS or amniocentesis)	Minimal maternal risk.	Limited by locus heterogeneity; CVS findings may require clarification by amniocentesis if placental mosaicism is suspected.
III: complex recessive	Severe skeletal dysplasias (28)	AR/AD (highly heterogeneous)	Multiple	ECS + confirmatory invasive prenatal diagnosis (Amniocentesis preferred)	Minimal maternal risk.	Severe phenotypes may appear on US long before a single molecular diagnosis is suspected; broad sequencing often required.
III: complex recessive	cTTP (29–31)	AR	*ADAMTS13*	ECS + Maternal ADAMTS13 monitoring + Plasma Exchange	Systemic Thrombotic Storm (triggered by physiological rise of vWF).	Fetus succumbs to uterine hypoxia; maternal acute multi-organ failure.
IV: blind spot	sJIA (32, 33)	Polygenic/complex	Multiple (IL-1, IL-6 pathways)	Non-invasive counseling + exclusionary WES (postnatal)	Minimal specific risk (unless on biologic therapies).	Entirely incapable of reliable prenatal prediction via genomic sequencing.

ACH, Achondroplasia; AD, Autosomal dominant; AR, autosomal recessive; cTTP, congenital thrombotic thrombocytopenic purpura; ECS, expanded carrier screening; FAP, familial adenomatous polyposis; NCL, neuronal ceroid lipofuscinosis; NF1, Neurofibromatosis type 1; NIPT-SGD, noninvasive prenatal testing for single-gene disorders; OI, osteogenesis imperfecta; PCD, primary ciliary dyskinesia; PGT-M, preimplantation genetic testing for monogenic disorders; sJIA, systemic juvenile idiopathic arthritis; US, ultrasound; VHL, von Hippel–Lindau Syndrome; WES, whole-exome sequencing.

Taken together, this archetype-based classification serves as a practical bridge between genomic complexity and real-world obstetric action. Rather than replacing disease-specific recommendations, it provides a higher-order clinical framework for aligning each category of genetic risk with the most appropriate timing and modality of intervention (Figures 1, 2).

## 3 Archetype I: Targeted mid-to-late trimester interception for *de novo* mutations

A substantial proportion of dominant genetic disorders arise from *de novo* germline mutations (7). In such cases, routine preconception carrier screening in apparently healthy parents is typically uninformative, and traditional pedigree-based counseling may fail to identify risk. Achondroplasia is a prototypical example of this clinical dilemma. As the most common skeletal dysplasia, achondroplasia (ACH) is usually caused by pathogenic variants in FGFR3, and most affected infants are born to unaffected parents (8). Advanced paternal age is an important clinical clue, and obstetricians should remain vigilant when evaluating such pregnancies (9, 10).

Routine ultrasound surveillance forms the first line of detection. A fetal femur length significantly below the expected percentile in the mid-to-late second trimester should prompt further evaluation (10). Conventional karyotyping and chromosomal microarray analysis are generally insufficient in this setting because the underlying defect is often a single pathogenic variant at a defined locus. Beyond achondroplasia, this archetype also includes other dominantly inherited conditions with frequent *de novo* occurrence, such as selected forms of osteogenesis imperfecta (11) and, in some clinical contexts, neurofibromatosis type 1 (12).

Targeted noninvasive prenatal testing for single-gene disorders has emerged as a complementary approach in selected high-risk scenarios (13, 14). Depending on platform and panel design, currently available assays may interrogate dozens to more than one hundred genes associated with dominant monogenic disorders; however, they do not detect all possible pathogenic variants within those genes and therefore should not be interpreted as comprehensive exclusion tests (13, 14). Pretest counseling should clearly explain assay scope, residual risk, and the need for confirmatory diagnostic testing after a positive screening result.

When a severe diagnosis is confirmed, counseling should remain non-directive and should include discussion of continuation of pregnancy with perinatal planning, as well as termination of pregnancy where legally permitted and consistent with parental values. In addition to facilitating reproductive decision-making, earlier molecular diagnosis may help the obstetric team optimize delivery planning and neonatal management.

## 4 Archetype II: Preconception source eradication for late-onset tumor syndromes

Certain genetic disorders exhibit no detectable abnormalities during fetal development. They manifest life-threatening conditions only in adulthood. Familial adenomatous polyposis (FAP) and von Hippel–Lindau (VHL) syndrome are prototypic examples. Affected individuals face a high lifetime risk of developing multiple malignancies across various organ systems (15, 16). Diagnosing fetal affection in the second trimester via amniocentesis places both the obstetrician and the family in an extremely difficult ethical position. Parents face the agonizing decision of whether to terminate a pregnancy carrying a structurally normal fetus (17).

The most humane and effective clinical intervention must shift significantly earlier to the preconception period. Couples with a clear family history require direct referral to reproductive endocrinology and genetics centers. Preimplantation genetic testing for monogenic disorders (PGT-M) enables the precise selection of unaffected embryos (18).

**Figure 3:**
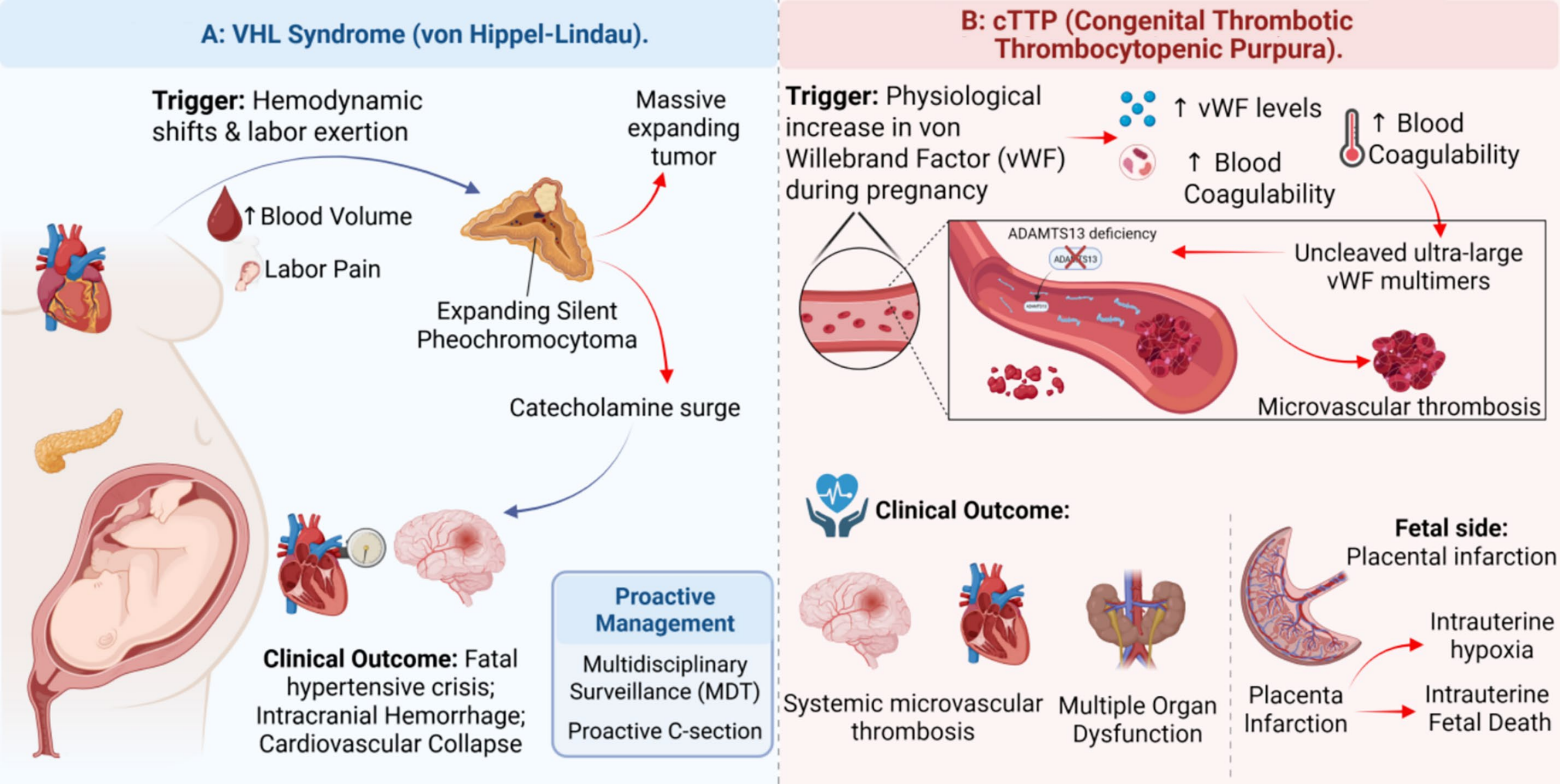
Pregnancy as a stress test: Maternal pathophysiological risks in specific genetic archetypes. Obstetric management extends beyond fetal screening to encompass severe maternal risks. **(A)** In VHL syndrome, the physiological demands of pregnancy and the exertion of labor can precipitate a fatal hypertensive crisis from an underlying, previously silent pheochromocytoma. **(B)** In cTTP, the dramatic gestational rise in von Willebrand factor (vWF) overwhelms the deficient ADAMTS13 enzyme, triggering systemic microvascular thrombosis. Both scenarios necessitate multidisciplinary maternal-fetal surveillance and proactive peripartum management.

The pregnant woman’s own safety remains paramount at this stage. Women with VHL syndrome face substantially elevated maternal mortality risks during pregnancy. The profound physiological and hemodynamic changes of gestation act as an extreme stress test. These changes can precipitate the emergence or rupture of previously silent pheochromocytomas (19). The intense pain and exertion of labor can trigger a fatal hypertensive crisis (Figure 3A). Cardiovascular, endocrine, and maternal-fetal medicine specialists must actively collaborate in antenatal and peripartum care.

## 5 Archetype III: Early defense and maternal protection for complex recessive diseases

Highly heterogeneous recessive disorders demand a more upstream and flexible defense strategy. Primary ciliary dyskinesia (PCD) and neuronal ceroid lipofuscinosis (NCL) exemplify this category, as both involve numerous dispersed pathogenic genes and cannot be adequately addressed by narrow targeted prenatal assays (20–22). In such settings, routine ultrasound may also be insufficient because fetal structural manifestations are often absent, subtle, or nonspecific (23, 24).

The implementation of expanded carrier screening (ECS) in the preconception period offers the most practical first-line strategy for these disorders (6, 25). If both partners are found to carry pathogenic variants in the same gene, definitive fetal diagnosis should be offered through invasive testing, including first-trimester chorionic villus sampling or mid-trimester amniocentesis. Because chorionic villus sampling analyzes placental tissue, interpretation may occasionally be complicated by confined placental mosaicism; in such situations, amniocentesis may be preferable because it more directly reflects the fetal genotype (26, 27).

Severe skeletal dysplasias should also be considered within this broader discussion of genetic heterogeneity. Unlike achondroplasia, which serves here as a paradigm of a recurrent and targetable *de novo* disorder, many prenatal skeletal phenotypes are genetically diverse and may require broader molecular strategies rather than single-gene testing alone (28). For these conditions, the combination of preconception risk assessment, ultrasound-triggered suspicion, invasive prenatal sampling, and phenotype-directed genomic testing may provide a more realistic diagnostic pathway.

Congenital thrombotic thrombocytopenic purpura (cTTP) deserves particular emphasis within this archetype because it poses a dual maternal-fetal threat. Pregnancy itself can precipitate acute disease activity through profound gestational changes in the balance between von Willebrand factor and ADAMTS13, thereby triggering life-threatening systemic microvascular thrombosis (Figure 3B). The fetus may suffer from placental insufficiency, growth restriction, or intrauterine demise, whereas the mother remains at risk of severe multiorgan injury (29). Obstetric teams should therefore closely monitor ADAMTS13 activity throughout pregnancy and coordinate management with hematologists prepared to initiate prophylactic or rescue plasma-based therapy when indicated (30, 31).

## 6 Archetype IV: Prediction blind spots and exclusionary diagnosis

Genetic sequencing technology is not a panacea for all medical challenges. Systemic juvenile idiopathic arthritis (sJIA) serves as an instructive counterexample (32). This condition arises from the complex interplay of multiple minor genetic variants and environmental triggers (33). Current high-throughput sequencing technologies remain entirely incapable of predicting such polygenic conditions prenatally. Ordering NIPT or amniocentesis to screen for sJIA is clinically inappropriate. Clinicians must actively decline such diagnostic requests to prevent medical mismanagement.

Obstetricians carry a profound ethical responsibility to communicate the limitations of current genomic technology transparently to families. A maternal family history including unexplained febrile episodes and severe arthritis warrants a carefully considered diagnostic approach. Clinicians may recommend whole-exome sequencing (WES), but primarily for exclusionary diagnosis. This approach helps rule out phenotypically similar autoinflammatory disorders with clear, detectable monogenic causes (e.g., HLH-related gene defects). Recognizing the boundaries of genomic technology reflects true clinical rigor. Pediatric rheumatologists can subsequently use these exclusionary results to plan appropriate postnatal surveillance.

## 7 Interdisciplinary collaboration and clinical outlook

Implementing this stratified prevention system requires seamless interdisciplinary collaboration. The widespread use of genomic sequencing introduces a pervasive clinical challenge into the obstetric clinic. Clinicians frequently encounter variants of uncertain significance (VUS). Communicating the nuances of these ambiguous findings to pregnant women without medical backgrounds is a formidable task (34). We must consistently adhere to the core principle of non-directive genetic counseling. Clinicians should provide objective data regarding current knowledge and estimated phenotypic probabilities. Families must then make autonomous reproductive decisions aligned with their personal values (35).

Obstetricians must abandon the inertia of one-size-fits-all screening when faced with a vast array of rare diseases. We propose a novel clinical paradigm grounded in underlying biological mechanisms. This framework employs mid-trimester noninvasive technology to intercept *de novo* mutations. It utilizes preconception embryo selection to halt the transmission of late-onset tumor syndromes. Expanded carrier screening identifies hidden, highly heterogeneous recessive risks. Finally, heightened maternal surveillance for specific high-risk pregnancies safeguards the mother’s life. This archetype-based system transcends the limitations of static single-disease catalogs. It offers a logically coherent, actionable decision-making blueprint for global obstetric practice. Precisely matching each genomic screening modality to its appropriate disease archetype forms the cornerstone of building an effective, modern defense against birth defects. Future practice guidelines from professional societies should consider integrating this archetype-based stratification to navigate the increasing complexity of prenatal genomic findings, ultimately paving the way for true precision obstetrics.
